# The genetic code as a hypothetical product of primordial viral evolution

**DOI:** 10.1038/s44298-026-00206-4

**Published:** 2026-06-18

**Authors:** Lev G. Nemchinov

**Affiliations:** https://ror.org/03b08sh51 grid.507312.2 U.S. Department of Agriculture, Agricultural Research Service, Molecular Plant Pathology Laboratory, Beltsville Agricultural Research Center, Beltsville, MD USA

**Keywords:** Biochemistry, Computational biology and bioinformatics, Evolution, Genetics, Molecular biology

## Abstract

The genetic code is a universal script for life on Earth in which the information is stored in a complex nonbinary base-4 system composed in groups of three. It is generally accepted that during the prebiotic RNA World primordial RNA sequences likely recruited early amino acids using specific number of bases, possibly triplets. There are several hypothetical scenarios regarding how it happened. However, the evolutionary basis and the underlying rationale for codon-specific amino acid assignments are yet to be determined. Among the very first entities on Earth were selfish RNA replicons that lacked a genetic code and could only catalyze their own replication. Supposedly, these primitive selfish elements were essentially unstable naked RNAs that required protective shells to survive. By forming an early protective shell (or capsid) from prebiotic amino acids linked to specific bases, they would not only facilitate the development of a rudimentary genetic code but become precursors to capsid-encoding virus-like structures. This viewpoint suggests that the requirement for genetic material to be encapsidated in a protective protein shell initiated the development of the genetic code, resulting in the formation of the first primitive, virus-like entities preceding the cellular life. The proposed model complements existing hypotheses of the code origin rather than replacing them.

## Introduction

The genetic code is a universal script for life, suggesting that all organisms originated from a single ancestor or “closely interbreeding population”^1^. In spite of six decades have elapsed since the best version of the code was presented in 1966^1,2^, the exact origin of the code remains a mystery. Several conflicting theories sought to explain the code’s origin: the stereochemical hypothesis proposed specific affinity between codons or anticodons and amino acids^3,4^; code adaptation or error-minimization hypothesis proposed that the genetic code adapts to reduce the damage caused by mutations^1,5–7^; coevolution hypothesis proposed that the prebiotic pathways of the genetic code coevolved with the enzymatic pathways of amino acids biosynthesis^8^; and the frozen accident theory suggested that in the “extreme form” codon-to-amino-acid assignments were entirely random and the genetic code is frozen as any change to it would be lethal^1^.

While any or all of these hypotheses could be correct, they hardly explain “Why are the codon assignments what they are?”^9^. In other words, at what point and for what reason did the chemical acids made of carbon, hydrogen, oxygen, nitrogen, and phosphorus atoms transformed into the universal, information-bearing “script” that directs the major processes of life? Was it, perhaps, the fact that primordial genetic elements in the “prebiotic soup”^10–12^ were just floating around in a particularly stimulating way that somehow triggered the onset of natural selection process leading to emergence of the genetic code? Or was it because of the thermodynamics principles influencing the relative abundances of the early amino acids and their correspondence to the composition of the first proteins at the time the genetic code originated^13^? Or were the early amino acids and perhaps the primitive genetic code or even the first organisms delivered by extraterrestrial sources^14–17^? Once again, none of these theories can be bluntly accepted or rejected, some due to the obvious lack of convincing experimental evidence.

Therefore, despite new, important details emerging since the first publications on the origin and structure of the genetic code^1,3,18^, the fundamental question asked by Woese^18^ “…why there exists this particular, unique, precise correspondence between amino acids and codons”, remains unanswered. Clearly, though, the question implies that not merely the mechanics and chemistry of the prebiotic interactions between polynucleotides and polyamino acids were crucial for the development of the primitive code and nascent translation system, but also the underlying rationale for all these specific associations. What evolutionary forces could drive these pairings?

## Main text

From an evolutionary perspective, among other things, these interactions could be attributed to the proliferation of some of the very first entities on Earth - parasitic genetic elements and selfish replicators^7,19,20^. The earliest parasitic replicons of the RNA World^21^ unlikely possessed the genetic code and could only catalyze their own replication nonenzymatically, using ribozyme^22^, or with the help of the replicase provided by the putative host RNA system^23^. Supposedly, they were short, single-stranded RNAs possessing secondary structural elements in the form of several small stem loops ^22^.

Along with the development of the more sophisticated replication systems, primordial “naked” genetic elements were likely attempting to compartmentalize and secure their molecules inside protective shells by evolving mechanisms to translate RNA. The initial encapsidation of RNA aptomers with amino acids (aa) might have been driven by varying affinities of specific aa-RNA interactions ^24,25^. If an RNA molecule contained multiple aa binding sites, the bound amino acids would likely be spatially organized, forming a peptide^25^ and eventually a proto-capsid structure. Once replicons were enclosed in protective shells, natural selection likely preserved these early pairings because they offered a selective advantage.

As a result, capsidless genetic replicons could eventually become predecessors of different classes of virus-like entities, capsid-encoding organisms^7^. Given that viral capsids are primarily assembled from multiple copies of a single protein, it is plausible to suggest that the process behind the development of the early genetic code began with the interactions between prebiotic amino acids and bases of the parasitic replicons that were essential to encode this singular protein. In fact, the ability of capsids to self-assemble around the associated nucleic acid is well-known^26^. Although this suggestion is contradictory to the hypothetical origin of viral capsid proteins from cellular ancestors^27^, it is in line with the “virus-first” theory^12,28^ and does not conflict with the definition of viruses as capsid-encoding organisms^29^. Indeed, it is both logical and feasible that the genetic code’s origin and evolution could have been driven by the need to instruct the synthesis of a single, simple protein resembling viral capsid. This assumption positions viruses, via their ancestral genetic replicons, at the very beginning of the genetic code.

The group of ancient protein structures potentially derived from the primitive genetic code could include, for instance, the highly conserved, compact single jelly-roll capsid fold prevalent in the non-enveloped icosahedral +RNA viruses infecting all major cellular life forms^7,30^. Beyond that, the jelly roll folds could have been preceded by the more primitive type of folding arrangements, well-conserved small β-barrels protein folds like Double-Psi Beta-Barrel (DPBB), found in viral RNA-dependent RNA polymerases (RdRp).

The DPBB fold is a six-stranded β-barrel that was likely among the first primordial protein structures synthesized by an ancient translational machinery^31,32^. The DPBB domain was recently reconstructed using only seven amino acids (Ala, Asp, Glu, Gly, Lys, Arg, Val), demonstrating that this ancient protein structure could have been produced by early translation systems with very limited genetic coding involved, GNN (N = any of the four nucleotides) and ARR (R = A or G)^31^. Interestingly, of this set of seven amino acids, at least five (Ala, Asp, Glu, Gly and Val) are considered prebiotic, that is, available abiotically rather than as a result of the genetic coding^9,33–35^. Prebiotic amino acids are thought to be involved in the earliest interactions with the polynucleotides under “primitive earth conditions”^18^. Also critical is the apparent nucleic acid-binding ability of the ancestral DPBB^31^, as it may imply its possible role in the formation of the ancient genetic code.

Thus, hypothetically, the emergence of the DPBB and jelly roll folds could stem from the further development of the ancestral encapsulated structures transitioning into more complex protein capsids and functional catalytic domains essential for the evolution of the primeval RNA self-replicons ^31^.

The encapsidated RNA replicons may have further developed the ability to synthesize simple peptides that could influence their structure and expand available functional space^36^, potentially resulting in the formation of RNA hairpins with anticodon-like nucleotides. Indeed, single-stranded RNA viruses often possess 3′-terminal tRNA-like structures, which are thought to be “fossils” of the RNA World and potential precursors to anticodons^37^. Similar to Yarus’s^24^ Direct-RNA-Template Theory, these ancestral anticodons could have then paired with the existing RNA triplets, aligning with (or adapting) the amino acid already bound to the RNA replicon and thus allowing binding sites to become coding sequences^24^. Besides, the smaller peptides originated from the primitive code or otherwise minimally-encoded and acting as protective sheath for RNA replicons, could have continued to evolve by repetition and accretion of their subdomains^38,39^, transitioning to more complex capsid-like envelopes preventing RNA from degradation.

Thus, assuming that capsidless selfish replicons were indeed ancestral to all other organisms, their immediate evolutionary advancement would be the formation of a rudimentary genetic code allowing them to produce vital for their survival capsid protein. This would essentially make them capsid-encoding, self-replicating primordial virus-like entities^9^, potentially serving as precursors to all cellular life forms ^20,40^.

Because electrostatic interactions between capsid proteins and RNA are essential for the assembly of modern viruses^41^, similar forces could have contributed to the formation and stability of the enveloped RNA replicons^42^. Furthermore, as only a few basic aa residues were found to be sufficient for a high-affinity binding of capsid protein with viral RNA to trigger virion assembly^43^, the earliest prebiotic associations that gave rise to the RNA encapsidation and the genetic code may have been driven by electrostatic interactions between a limited subset of biophysically similar amino acids and RNA aptamers ^42^.

Although the evolutionary drive to encapsulate parasitic replicons within protein shells might explain the origin and specificity of the first genetic code, which of the tentative mechanisms mentioned above would allow for its formation and evolution in this direction? Regardless of what process took place^1,3–5,8,41,42^, the very first interactions of prebiotic amino acids and code-less genetic elements likely occurred through binding to one another in part due to their abundance and proximity in the “prebiotic milieu”^18^. Assuming that the primary role in these interactions belonged to the early replicating oligonucleotides while amino acids were utilized as cofactors supporting primitive ribozymes^44^, it cannot be ruled out that recruitment of the prebiotic amino acids had also been selectively directed toward protection and stabilizing of the naked RNA chains. Moreover, the first RNA replicons could also have been selected for their ability to accumulate amino acids and form encapsidated structures. Indeed “We shall argue that by far the most likely step was that these primitive amino acids spread all over the code until almost all the triplets represented one or other of them” ^1^.

Subsequently, these early specific associations between short chains of amino acids and RNA that have created a protective envelope around primitive RNA molecules enabled a rudimentary genetic code to emerge.

Given this, a valid question arises about the possible role of the primitive tRNAs (proto-tRNAs) in the origin of the genetic code^1^ through ability of the first amino acids to interact with the evolving tRNA anticodons^45,46^. As mentioned above, the viewpoint proposes that the genetic code initiated by the encapsidation of the early RNA replicons, predated the emergence of tRNA-like minihelices^47,48^. This is because the evolution of the proto-tRNAs required ribozyme-based mechanisms to generate RNA repeats and inverted repeats^48^ and the primitive aptameric replicons already had various ribozyme activities^49^. Therefore, the viewpoint predicts that the anticodons and the proto-tRNAs may have emerged from the primordial, cognate triplets of the primitive genetic code that was already established in capsid-bearing RNA structures. This aligns with the findings by Rodin et al^50^. who suggested that primordial tRNAs, tRNA-aminoacylating ribozymes, and the subsequent translation machinery evolved to adapt to the already defined genetic code, rather than the reverse.

**Fig. 1:**
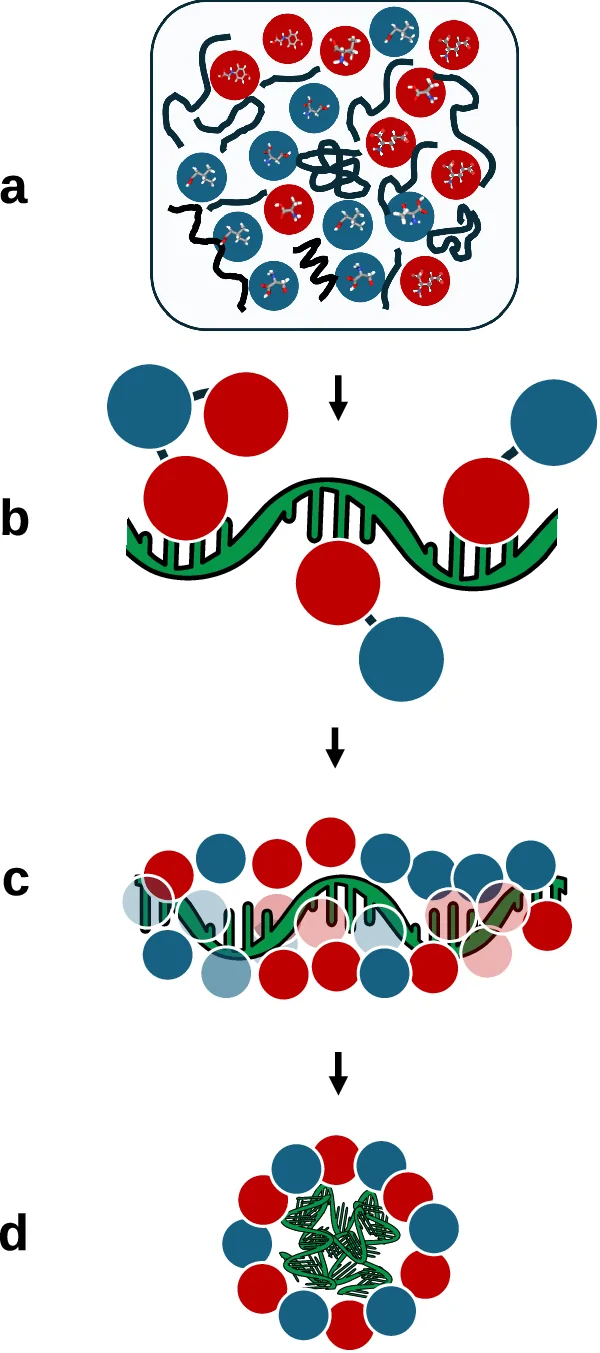
A model for the stepwise emergence of the genetic code. The figure represents a simplified conceptual scenario and should not be interpreted as a detailed mechanistic reconstruction. **a** Initial binding of prebiotic amino acids to primordial genetic replicons in the RNA World environment; **b** Establishing specific interactions between amino acids and RNA sequences; **c** development of the rudimentary genetic code as a source of information for the protective envelope; and **d** emergence of the viral-like particles. Blue and red circles represent hydrophilic and hydrophobic amino acids; black lines represent primordial genetic elements; oversimplified RNA molecule is shown in green.

It is plausible that primordial, encapsidated, ribonucleoprotein structures were reminiscent of archaic virus-like particles, precursors to modern viruses. Therefore, the chain of events involving (a) initial binding of prebiotic amino acid to selfish genetic elements to protect and stabilize them; (b) establishing specific interactions between amino acids and RNA sequences; (c) development of the rudimentary genetic code as a source of information for the protective envelope; and (d) emergence of the viral-like particles, may offer a feasible explanation for the origin of the genetic code (Fig. 1).

Simply put, the need for a protective protein/amino acid coat for early genetic material drove the early development of the genetic code, thus leading to the emergence of the first virus-like entities. This implies that the first information contained in the primitive genetic code to be successfully passed on was associated with the protein shells of archaic virus-like structures.

Setting aside the putative mechanisms allowing the original interactions between RNA replicons and amino acids^3–6,8,9,41,42^, this viewpoint focuses on the primary driver, an obvious evolutionary pressure on the selfish genetic elements to adapt by developing the early genetic code for their protective protein shells. Predictably, these first capsid-encoding units transitioned into virus-like forms^7^, picking up all components they needed from surrounding environment ^51^ to start their assembly lines and eventually paving the way for cellular life forms ^52^.

## Conclusion

It is believed that primary amino acids and short peptides likely bound to primitive RNA molecules to stabilize them against degradation^53–55^. It was also suggested that primordial genetic elements evolved into viruses^7,51^ and that viruses might have preceded cellular life^12^. Therefore, the two main pillars the viewpoint presented in this article is built upon are not new, having been known and/or proposed before.

What appears to be novel, however, is the alternative interpretation of these theoretical frameworks: early interactions of the prebiotic amino acids with self-replicons could have sparked encapsulation of the primitive RNA to stabilize and protect it and these natural associations prompted the development of the rudimentary genetic code. Thus, the evolutionary force for the emergence of the genetic code was the necessity of forming a protective protein shell around primordial genetic replicons. Accordingly, if the rudimentary genetic code contained instructions for synthesizing protective shells and capsid-encoding replicons evolved into modern viruses, it is accurate to say that the origin of the code was primarily driven by viral evolution.

Even if the proposed model only represents a possible selective pressure shaping the early code and does not fully explain the precise transition from amino acid/RNA interactions to specific codon–amino acid assignments, it nonetheless suggests that the origins of the genetic code and viruses should be viewed as inseparable co-evolutionary processes.

## Data Availability

No datasets were generated or analyzed during the current study.
